# Poly[[aqua­tris­(μ_3_-hexa­methyl­ene­tetra­mine-κ^3^ *N*,*N*′,*N*′′)tris­(*p*-toluene­sulfonato-κ*O*)tris­ilver(I)] trihydrate]

**DOI:** 10.1107/S1600536810048567

**Published:** 2010-11-27

**Authors:** Hua Wu, Meng-Xiang Shang, Shao-Ping ShangGuan

**Affiliations:** a Heilongjiang Agricultural Vocational and Technical College, JiaMuSi, HeiLongJiang 154007, People’s Republic of China

## Abstract

There are three Ag^I^ cations, three *p*-toluene­sulfonate (pts) anions, three hexa­methyl­ene­tetra­mine (hmt) mol­ecules and four water mol­ecules in the asymmetric unit of the title coordination polymer, {[Ag_3_(C_7_H_7_O_3_S)_3_(C_6_H_12_N_4_)_3_(H_2_O)]·3H_2_O}_*n*_. Two of the pts anions show positional disorder of their O atoms in 0.60:0.40 and 0.50:0.50 ratios. The Ag^I^ ion is coordinated by three hmt mol­ecules in an approximate trigonal–planar AgN_3_ arrangement. In each case, longer Ag—O bonds to a water mol­ecule and a pts anion complete a distorted trigonal–bipyramidal AgN_3_O_2_ geometry for the metal ion. In the crystal, the bridging hmt mol­ecules and pts ions generate a wave-like layer parallel to (001) and O—H⋯O hydrogen-bonding inter­actions consolidate the packing.

## Related literature

For background to metal-coordination networks containing both sulfonate anions and N-bonded ligands, see: Côté & Shimizu (2003 ▶); Zhang *et al.* (2001 ▶).
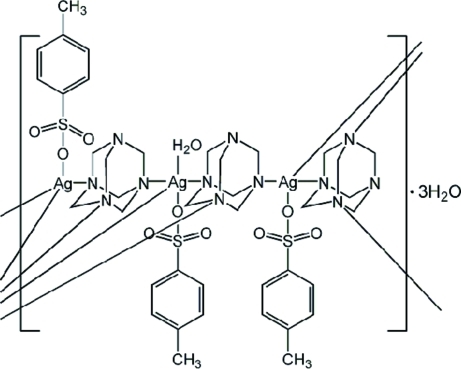


## Experimental

### 

#### Crystal data

[Ag_3_(C_7_H_7_O_3_S)_3_(C_6_H_12_N_4_)_3_(H_2_O)]·3H_2_O
*M* *_r_* = 1329.82
Monoclinic, 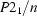

*a* = 17.3181 (5) Å
*b* = 10.7028 (3) Å
*c* = 26.9110 (11) Å
β = 95.657 (3)°
*V* = 4963.7 (3) Å^3^
*Z* = 4
Mo *K*α radiation
μ = 1.37 mm^−1^
*T* = 293 K
0.30 × 0.25 × 0.22 mm

#### Data collection

Oxford Diffraction Gemini R Ultra diffractometer
Absorption correction: multi-scan (*CrysAlis RED*; Oxford Diffraction, 2006 ▶) *T*_min_ = 0.672, *T*_max_ = 0.728
21413 measured reflections
11446 independent reflections
7890 reflections with *I* > 2σ(*I*)
*R*_int_ = 0.021

#### Refinement

*R*[*F* ^2^ > 2σ(*F* ^2^)] = 0.038
*wR*(*F* ^2^) = 0.097
*S* = 0.99
11446 reflections
650 parameters
12 restraints
H atoms treated by a mixture of independent and constrained refinement
Δρ_max_ = 0.90 e Å^−3^
Δρ_min_ = −1.05 e Å^−3^

### 

Data collection: *CrysAlis CCD* (Oxford Diffraction, 2006 ▶); cell refinement: *CrysAlis CCD*; data reduction: *CrysAlis RED* (Oxford Diffraction, 2006 ▶); program(s) used to solve structure: *SHELXS97* (Sheldrick, 2008 ▶); program(s) used to refine structure: *SHELXL97* (Sheldrick, 2008 ▶); molecular graphics: *SHELXTL-Plus* (Sheldrick, 2008 ▶); software used to prepare material for publication: *SHELXL97*.

## Supplementary Material

Crystal structure: contains datablocks I, global. DOI: 10.1107/S1600536810048567/hb5739sup1.cif

Structure factors: contains datablocks I. DOI: 10.1107/S1600536810048567/hb5739Isup2.hkl

Additional supplementary materials:  crystallographic information; 3D view; checkCIF report

## Figures and Tables

**Table 1:** Selected bond lengths (Å)

Ag1—N1	2.362 (2)
Ag1—N9^i^	2.367 (3)
Ag1—N10^ii^	2.388 (3)
Ag2—N5	2.347 (2)
Ag2—N7^ii^	2.365 (3)
Ag2—N4	2.374 (2)
Ag3—N3^ii^	2.315 (3)
Ag3—N8	2.358 (3)
Ag3—N11	2.394 (2)

Symmetry codes: (i) 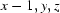; (ii) 
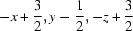
.

**Table 2:** Hydrogen-bond geometry (Å, °)

*D*—H⋯*A*	*D*—H	H⋯*A*	*D*⋯*A*	*D*—H⋯*A*
O1*W*—H1*WA*⋯O8^ii^	0.87 (2)	1.92 (2)	2.782 (5)	175 (4)
O1*W*—H1*WB*⋯O6^ii^	0.82 (2)	2.10 (2)	2.907 (4)	166 (4)
O2*W*—H2*WA*⋯O5^iii^	0.83 (2)	2.16 (2)	2.958 (4)	163 (4)
O2*W*—H2*WB*⋯O1^iv^	0.84 (2)	1.83 (3)	2.612 (6)	155 (4)
O3*W*—H3*WA*⋯O2^v^	0.80 (2)	2.45 (3)	3.096 (7)	139 (4)
O3*W*—H3*WB*⋯O5	0.80 (2)	2.15 (2)	2.908 (5)	160 (4)
O4*W*—H4*WB*⋯O7^iii^	0.88 (2)	1.99 (3)	2.838 (7)	160 (4)

Symmetry codes: (ii) 
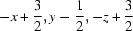
; (iii) 
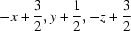
; (iv) 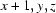; (v) 
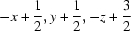
.

**Fig. 1.**
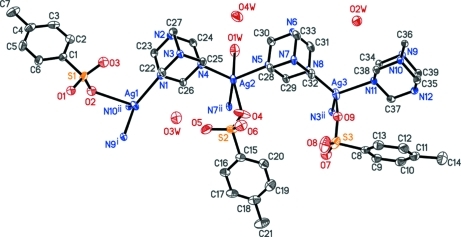
The molecular structure of the title compound with displacement ellipsoids drawn at the 30% probability level. [Symmetry codes: (i) x - 1, y, z; (ii) -x + 3/2, y - 1/2, -z + 3/2].

**Fig. 2.**
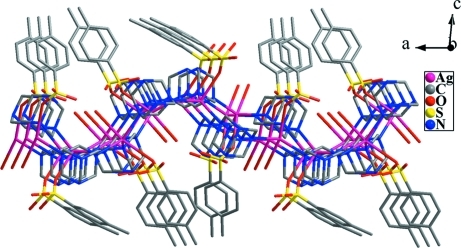
The two dimensional wave like layer of the title compound.

## supplementary crystallographic information

### Comment

Metal sulfonate complexes modified by different nitrogen-containing secondary ligands have been of great interest due to their abilities to form various structures, possible extended supramolecular system and good properties (Côté & Shimizu, 2003). Currently, there are some Ag^I^ sulfonate coordination polymers building from hexamethylenetetramine ligand because of its multidentate coordination mode (Zhang *et al.*, 2001).

In the crystal structure of the title compound, C_39_H_65_Ag_3_N_12_O_13_S_3_, there are three Ag^I^ cations, three *p*-toluenesulfonate anions, three hexamethylenetetramine and four water molecules (Fig. 1). Ag1 cation is four-coordinated by three N atoms from three different hexamethylenetetramine ligands [Ag1—N1 = 2.362 (2), Ag1—N9^i^ = 2.367 (2), and Ag1—N10^ii^ = 2.388 (3)] and one O atom from one *p*-toluenesulfonate ligand [Ag1—O = 2.644 (6) Å] in a distorted tetrahedral coordination geometry. Ag2 cation is five-coordinated by three N atoms from three different hexamethylenetetramine ligands [Ag2—N5 = 2.347 (2), Ag2—N4 = 2.374 (2) and Ag2—N7^ii^ = 2.365 (2) Å], one O atom from one *p*-toluenesulfonate ligand and one water molecule [Ag2—O4 = 2.622 (3) and Ag2—O1W = 2.622 (4) Å] in a trigonalbiyramid coordination geometry. Ag3 cation is also four-coordinated by three N atoms from hexamethylenetetramine ligands [Ag3—N3^ii^ = 2.315 (3), Ag3—N8 = 2.358 (3), and Ag3—N11 = 2.394 (2) Å] and one *p*-toluenesulfonate ligand [Ag3—O9 = 2.438 (5) Å] in a distorted tetrahedral coordination geometry. The Ag^I^ cations are bridged by hexamethylenetetramine molecules in tridentate modes to generate a two dimensional wave like layer with the *p*-toluenesulfonate ligands hanged up and down (Fig. 2). The intermoleclar hydrogen bonding interactions consolidate the layer.

### Experimental

An aqueous solution (10 ml) of *p*-toluenesulfonic acid (0.038 g, 0.3 mmol) was added to solid Ag_2_CO_3_ (0.041 g, 0.15 mmol) and stirred for several stirred for several minutes until no further CO_2_ was given off; and hexamethylenetetramine (0.028 g, 0.2 mmol) was added in. The white precipitate was dissolved by dropwise addition of an aqueous solution of NH_3_ (14 *M*).  Colourless blocks were obtained by evaporation of the solution for several days at room temperature.

### Refinement

The disordered O atoms (O1, O2, O3, O7, and O9) of *p*-toluenesulfonate ligands split over two sites with a total ocuupancy of 1. C–bound H–atoms were geometrically positioned (C—H 0.93 Å) and refined using a riding model, with *U*_iso_ = 1.2*U*_eq_ (C). The water H atoms were located in a difference Fourier map and refined with *U*_iso_(H)= 1.5Ueq(O).

### Crystal data

**Table:**

[Ag_3_(C_7_H_7_O_3_S)_3_(C_6_H_12_N_4_)_3_(H_2_O)]·3H_2_O	*F*(000) = 2704
*M**_r_* = 1329.82	*D*_x_ = 1.779 Mg m^−^^3^
Monoclinic, *P*2_1_/*n*	Mo *K*α radiation, λ = 0.71073 Å
Hall symbol: -P 2yn	Cell parameters from 11446 reflections
*a* = 17.3181 (5) Å	θ = 3.0–29.3°
*b* = 10.7028 (3) Å	µ = 1.37 mm^−^^1^
*c* = 26.9110 (11) Å	*T* = 293 K
β = 95.657 (3)°	Block, colorless
*V* = 4963.7 (3) Å^3^	0.30 × 0.25 × 0.22 mm
*Z* = 4	

### Data collection

**Table:**

Oxford Diffraction Gemini R Ultra diffractometer	11446 independent reflections
Radiation source: fine-focus sealed tube	7890 reflections with *I* > 2σ(*I*)
graphite	*R*_int_ = 0.021
Detector resolution: 10.0 pixels mm^-1^	θ_max_ = 29.3°, θ_min_ = 3.0°
ω scans	*h* = −23→19
Absorption correction: multi-scan (*CrysAlis RED*; Oxford Diffraction, 2006)	*k* = −13→8
*T*_min_ = 0.672, *T*_max_ = 0.728	*l* = −24→36
21413 measured reflections	

### Refinement

**Table:**

Refinement on *F*^2^	Primary atom site location: structure-invariant direct methods
Least-squares matrix: full	Secondary atom site location: difference Fourier map
*R*[*F*^2^ > 2σ(*F*^2^)] = 0.038	Hydrogen site location: inferred from neighbouring sites
*wR*(*F*^2^) = 0.097	H atoms treated by a mixture of independent and constrained refinement
*S* = 0.99	*w* = 1/[σ^2^(*F*_o_^2^) + (0.054*P*)^2^] where *P* = (*F*_o_^2^ + 2*F*_c_^2^)/3
11446 reflections	(Δ/σ)_max_ = 0.002
650 parameters	Δρ_max_ = 0.90 e Å^−^^3^
12 restraints	Δρ_min_ = −1.05 e Å^−^^3^

### Special details

**Table:**

Geometry. All e.s.d.'s (except the e.s.d. in the dihedral angle between two l.s. planes) are estimated using the full covariance matrix. The cell e.s.d.'s are taken into account individually in the estimation of e.s.d.'s in distances, angles and torsion angles; correlations between e.s.d.'s in cell parameters are only used when they are defined by crystal symmetry. An approximate (isotropic) treatment of cell e.s.d.'s is used for estimating e.s.d.'s involving l.s. planes.
Refinement. Refinement of *F*^2^ against ALL reflections. The weighted *R*-factor *wR* and goodness of fit *S* are based on *F*^2^, conventional *R*-factors *R* are based on *F*, with *F* set to zero for negative *F*^2^. The threshold expression of *F*^2^ > σ(*F*^2^) is used only for calculating *R*-factors(gt) *etc*. and is not relevant to the choice of reflections for refinement. *R*-factors based on *F*^2^ are statistically about twice as large as those based on *F*, and *R*- factors based on ALL data will be even larger.

### Fractional atomic coordinates and isotropic or equivalent isotropic displacement parameters (Å^2^)

**Table:**

	*x*	*y*	*z*	*U*_iso_*/*U*_eq_	Occ. (<1)
Ag1	0.314657 (14)	0.08332 (2)	0.713161 (11)	0.03692 (8)	
Ag2	0.667686 (12)	0.06405 (2)	0.717069 (11)	0.03702 (8)	
Ag3	1.009181 (14)	0.07397 (3)	0.816707 (13)	0.04627 (9)	
C1	0.10904 (18)	0.1984 (3)	0.57691 (13)	0.0351 (8)	
C2	0.1425 (2)	0.1533 (4)	0.53604 (14)	0.0463 (9)	
H2	0.1955	0.1370	0.5383	0.056*	
C3	0.0967 (3)	0.1325 (4)	0.49154 (15)	0.0538 (11)	
H3	0.1199	0.1021	0.4642	0.065*	
C4	0.0182 (3)	0.1555 (4)	0.48654 (15)	0.0530 (11)	
C5	−0.0141 (2)	0.1996 (4)	0.52791 (17)	0.0565 (11)	
H5	−0.0672	0.2149	0.5256	0.068*	
C6	0.0298 (2)	0.2218 (4)	0.57274 (15)	0.0469 (10)	
H6	0.0064	0.2523	0.6000	0.056*	
C7	−0.0295 (3)	0.1341 (5)	0.43795 (17)	0.0801 (16)	
H7A	−0.0827	0.1542	0.4414	0.120*	
H7B	−0.0257	0.0480	0.4285	0.120*	
H7C	−0.0108	0.1863	0.4127	0.120*	
C8	1.03065 (19)	0.2536 (4)	0.96495 (12)	0.0377 (8)	
C9	1.0778 (2)	0.1596 (4)	0.98513 (14)	0.0471 (10)	
H9	1.0612	0.0769	0.9830	0.057*	
C10	1.1501 (2)	0.1887 (5)	1.00856 (15)	0.0591 (12)	
H10	1.1816	0.1252	1.0227	0.071*	
C11	1.1766 (2)	0.3103 (6)	1.01136 (16)	0.0645 (13)	
C12	1.1281 (3)	0.4044 (5)	0.99292 (17)	0.0628 (12)	
H12	1.1443	0.4871	0.9960	0.075*	
C13	1.0550 (2)	0.3767 (4)	0.96963 (15)	0.0506 (10)	
H13	1.0223	0.4407	0.9572	0.061*	
C14	1.2587 (3)	0.3412 (8)	1.0342 (2)	0.105 (2)	
H14A	1.2669	0.4298	1.0328	0.157*	
H14B	1.2957	0.2992	1.0157	0.157*	
H14C	1.2651	0.3140	1.0683	0.157*	
C15	0.6257 (2)	0.1507 (3)	0.89555 (14)	0.0399 (8)	
C16	0.5692 (2)	0.0757 (4)	0.91222 (15)	0.0472 (9)	
H16	0.5222	0.0651	0.8928	0.057*	
C17	0.5822 (3)	0.0165 (4)	0.95755 (16)	0.0578 (11)	
H17	0.5434	−0.0338	0.9685	0.069*	
C18	0.6505 (3)	0.0297 (4)	0.98689 (17)	0.0608 (12)	
C19	0.7078 (3)	0.1047 (5)	0.96987 (19)	0.0638 (12)	
H19	0.7547	0.1148	0.9894	0.077*	
C20	0.6959 (2)	0.1644 (4)	0.92434 (16)	0.0514 (10)	
H20	0.7349	0.2136	0.9131	0.062*	
C21	0.6636 (4)	−0.0304 (6)	1.03776 (19)	0.0922 (18)	
H21A	0.7148	−0.0104	1.0526	0.138*	
H21B	0.6585	−0.1194	1.0344	0.138*	
H21C	0.6259	0.0003	1.0586	0.138*	
C22	0.42034 (16)	0.3050 (3)	0.68223 (13)	0.0289 (7)	
H22A	0.4236	0.3202	0.7179	0.035*	
H22B	0.3727	0.3426	0.6672	0.035*	
C23	0.41350 (18)	0.1495 (3)	0.61824 (13)	0.0340 (8)	
H23A	0.4117	0.0605	0.6113	0.041*	
H23B	0.3661	0.1866	0.6026	0.041*	
C24	0.55120 (18)	0.1488 (3)	0.61999 (13)	0.0369 (8)	
H24A	0.5952	0.1855	0.6057	0.044*	
H24B	0.5510	0.0598	0.6131	0.044*	
C25	0.55931 (16)	0.3047 (3)	0.68477 (13)	0.0290 (7)	
H25A	0.6039	0.3428	0.6716	0.035*	
H25B	0.5637	0.3189	0.7205	0.035*	
C26	0.49138 (16)	0.1145 (3)	0.69615 (13)	0.0302 (7)	
H26A	0.4958	0.1293	0.7319	0.036*	
H26B	0.4907	0.0249	0.6908	0.036*	
C27	0.48138 (19)	0.3383 (3)	0.60693 (13)	0.0386 (8)	
H27A	0.4345	0.3764	0.5910	0.046*	
H27B	0.5253	0.3755	0.5927	0.046*	
C28	0.77641 (16)	0.2991 (3)	0.73890 (13)	0.0299 (7)	
H28A	0.7571	0.2935	0.7715	0.036*	
H28B	0.7382	0.3438	0.7169	0.036*	
C29	0.84460 (16)	0.1052 (3)	0.75281 (14)	0.0316 (7)	
H29A	0.8264	0.0977	0.7856	0.038*	
H29B	0.8515	0.0216	0.7400	0.038*	
C30	0.81468 (19)	0.1839 (3)	0.66969 (14)	0.0384 (8)	
H30A	0.8211	0.1012	0.6559	0.046*	
H30B	0.7767	0.2283	0.6474	0.046*	
C31	0.94465 (18)	0.1821 (3)	0.70622 (15)	0.0424 (9)	
H31A	0.9943	0.2245	0.7080	0.051*	
H31B	0.9515	0.0990	0.6929	0.051*	
C32	0.90727 (17)	0.2980 (3)	0.77631 (14)	0.0347 (8)	
H32A	0.9563	0.3426	0.7797	0.042*	
H32B	0.8892	0.2916	0.8092	0.042*	
C33	0.87806 (19)	0.3752 (3)	0.69322 (14)	0.0385 (8)	
H33A	0.8409	0.4213	0.6709	0.046*	
H33B	0.9270	0.4199	0.6952	0.046*	
C34	1.17915 (16)	0.1141 (3)	0.78134 (13)	0.0294 (7)	
H34A	1.1504	0.1247	0.7488	0.035*	
H34B	1.1854	0.0252	0.7875	0.035*	
C35	1.29800 (17)	0.1580 (3)	0.83154 (13)	0.0333 (8)	
H35A	1.3052	0.0697	0.8388	0.040*	
H35B	1.3489	0.1960	0.8322	0.040*	
C36	1.24515 (17)	0.3086 (3)	0.77130 (12)	0.0267 (7)	
H36A	1.2953	0.3488	0.7711	0.032*	
H36B	1.2170	0.3202	0.7387	0.032*	
C37	1.18014 (18)	0.1554 (3)	0.86903 (13)	0.0340 (8)	
H37A	1.1516	0.1921	0.8946	0.041*	
H37B	1.1871	0.0672	0.8765	0.041*	
C38	1.12622 (16)	0.3044 (3)	0.80919 (13)	0.0297 (7)	
H38A	1.0974	0.3154	0.7767	0.036*	
H38B	1.0966	0.3426	0.8339	0.036*	
C39	1.24502 (18)	0.3481 (3)	0.85897 (13)	0.0334 (8)	
H39A	1.2953	0.3883	0.8597	0.040*	
H39B	1.2170	0.3865	0.8845	0.040*	
O1'	0.1480 (4)	0.3411 (7)	0.6499 (3)	0.0744 (19)*	0.50
O1	0.1172 (3)	0.2996 (5)	0.66542 (19)	0.0419 (12)*	0.50
O2'	0.1309 (5)	0.1218 (9)	0.6678 (3)	0.108 (3)*	0.50
O2	0.1813 (3)	0.1080 (5)	0.6571 (2)	0.0513 (14)*	0.50
O1W	0.70784 (18)	−0.0600 (3)	0.63965 (14)	0.0704 (10)	
O3'	0.2382 (4)	0.1850 (9)	0.6303 (3)	0.096 (2)*	0.50
O3	0.2377 (3)	0.2902 (6)	0.6240 (2)	0.0566 (14)*	0.50
O2W	1.06791 (15)	0.4929 (3)	0.71250 (11)	0.0543 (7)	
O4	0.6619 (2)	0.1634 (3)	0.80569 (12)	0.0881 (12)	
O3W	0.4316 (2)	0.4268 (4)	0.79689 (14)	0.0760 (10)	
O5	0.53010 (19)	0.2102 (3)	0.81959 (11)	0.0735 (10)	
O4W	0.6873 (2)	0.4746 (4)	0.62992 (15)	0.0830 (11)	
O6	0.63193 (17)	0.3562 (3)	0.84465 (11)	0.0600 (8)	
O7'	0.8959 (6)	0.1405 (12)	0.9688 (4)	0.109 (3)*	0.40
O7	0.9210 (3)	0.0947 (5)	0.9404 (2)	0.0670 (14)*	0.60
O8	0.89252 (18)	0.3193 (4)	0.93192 (15)	0.0903 (12)	
O9	0.9709 (3)	0.2271 (5)	0.8767 (2)	0.0475 (13)*	0.50
O9'	0.9462 (6)	0.1437 (11)	0.8920 (4)	0.124 (3)*	0.50
S1	0.16466 (5)	0.22358 (10)	0.63466 (4)	0.0441 (2)	
S2	0.61121 (6)	0.22628 (9)	0.83691 (4)	0.0481 (2)	
S3	0.94276 (6)	0.21884 (11)	0.92946 (5)	0.0599 (3)	
N1	0.41778 (13)	0.1693 (2)	0.67309 (10)	0.0260 (6)	
N2	0.47982 (16)	0.2043 (3)	0.59677 (11)	0.0382 (7)	
N3	0.48729 (13)	0.3647 (2)	0.66152 (10)	0.0280 (6)	
N4	0.55957 (13)	0.1691 (2)	0.67493 (10)	0.0262 (6)	
N5	0.78586 (13)	0.1722 (2)	0.71920 (10)	0.0280 (6)	
N6	0.88871 (15)	0.2504 (3)	0.67246 (11)	0.0395 (7)	
N7	0.85025 (13)	0.3702 (2)	0.74346 (11)	0.0298 (6)	
N8	0.91990 (13)	0.1713 (2)	0.75711 (11)	0.0331 (7)	
N9	1.25674 (13)	0.1725 (2)	0.78090 (10)	0.0258 (6)	
N10	1.20165 (13)	0.3685 (2)	0.80957 (10)	0.0253 (5)	
N11	1.13457 (13)	0.1694 (2)	0.81992 (10)	0.0276 (6)	
N12	1.25592 (14)	0.2151 (3)	0.87016 (10)	0.0328 (6)	
H1WA	0.6786 (17)	−0.097 (4)	0.6160 (12)	0.049*	
H1WB	0.7504 (15)	−0.094 (4)	0.6411 (14)	0.049*	
H2WA	1.042 (2)	0.549 (3)	0.6976 (14)	0.049*	
H2WB	1.088 (2)	0.448 (3)	0.6915 (13)	0.049*	
H3WA	0.425 (2)	0.485 (3)	0.8145 (14)	0.049*	
H3WB	0.460 (2)	0.375 (3)	0.8101 (15)	0.049*	
H4WA	0.7258 (14)	0.504 (4)	0.6231 (15)	0.049*	
H4WB	0.6512 (16)	0.522 (4)	0.6144 (14)	0.049*	

### Atomic displacement parameters (Å^2^)

**Table:**

	*U*^11^	*U*^22^	*U*^33^	*U*^12^	*U*^13^	*U*^23^
Ag1	0.03300 (13)	0.02596 (14)	0.05419 (18)	−0.00300 (10)	0.01635 (12)	−0.00403 (12)
Ag2	0.02446 (12)	0.02352 (13)	0.06195 (19)	−0.00263 (9)	−0.00151 (11)	0.00299 (13)
Ag3	0.03051 (13)	0.02299 (14)	0.0846 (2)	0.00125 (10)	0.00192 (13)	0.00676 (14)
C1	0.0398 (17)	0.0307 (19)	0.034 (2)	0.0014 (14)	−0.0005 (15)	0.0044 (16)
C2	0.048 (2)	0.045 (2)	0.046 (2)	−0.0009 (18)	0.0078 (18)	−0.0004 (19)
C3	0.082 (3)	0.047 (3)	0.034 (2)	−0.019 (2)	0.012 (2)	−0.0011 (19)
C4	0.074 (3)	0.037 (2)	0.045 (3)	−0.019 (2)	−0.012 (2)	0.0112 (19)
C5	0.041 (2)	0.063 (3)	0.062 (3)	−0.0016 (19)	−0.014 (2)	0.005 (2)
C6	0.0402 (18)	0.053 (3)	0.046 (2)	0.0076 (17)	−0.0029 (17)	−0.001 (2)
C7	0.113 (4)	0.068 (4)	0.052 (3)	−0.034 (3)	−0.027 (3)	0.017 (3)
C8	0.0446 (18)	0.041 (2)	0.0289 (19)	0.0084 (16)	0.0097 (15)	0.0040 (17)
C9	0.050 (2)	0.051 (3)	0.041 (2)	0.0123 (18)	0.0073 (18)	0.0049 (19)
C10	0.054 (2)	0.082 (4)	0.042 (2)	0.026 (2)	0.0055 (19)	0.010 (2)
C11	0.053 (2)	0.101 (4)	0.040 (3)	0.002 (3)	0.006 (2)	0.001 (3)
C12	0.069 (3)	0.063 (3)	0.057 (3)	−0.020 (2)	0.012 (2)	0.000 (2)
C13	0.057 (2)	0.045 (2)	0.050 (2)	0.0084 (19)	0.0067 (19)	0.009 (2)
C14	0.070 (3)	0.159 (7)	0.083 (4)	−0.020 (4)	−0.004 (3)	−0.016 (4)
C15	0.052 (2)	0.0293 (19)	0.040 (2)	0.0067 (16)	0.0141 (17)	−0.0023 (17)
C16	0.060 (2)	0.041 (2)	0.041 (2)	−0.0041 (18)	0.0108 (18)	0.0017 (19)
C17	0.084 (3)	0.045 (3)	0.047 (3)	0.002 (2)	0.018 (2)	0.011 (2)
C18	0.089 (3)	0.046 (3)	0.048 (3)	0.026 (2)	0.013 (2)	0.008 (2)
C19	0.068 (3)	0.055 (3)	0.066 (3)	0.022 (2)	−0.008 (2)	0.000 (2)
C20	0.051 (2)	0.046 (2)	0.059 (3)	0.0081 (18)	0.013 (2)	0.002 (2)
C21	0.141 (5)	0.075 (4)	0.058 (3)	0.022 (4)	0.001 (3)	0.029 (3)
C22	0.0247 (14)	0.0204 (16)	0.043 (2)	0.0010 (12)	0.0083 (14)	−0.0054 (14)
C23	0.0317 (16)	0.0312 (19)	0.038 (2)	−0.0032 (14)	−0.0018 (15)	−0.0071 (16)
C24	0.0321 (16)	0.036 (2)	0.045 (2)	0.0018 (14)	0.0124 (15)	−0.0059 (17)
C25	0.0249 (14)	0.0228 (16)	0.0388 (19)	−0.0020 (12)	0.0007 (13)	0.0013 (15)
C26	0.0277 (15)	0.0214 (16)	0.042 (2)	−0.0001 (12)	0.0031 (14)	0.0052 (15)
C27	0.0376 (17)	0.036 (2)	0.041 (2)	−0.0004 (15)	0.0019 (16)	0.0133 (17)
C28	0.0247 (14)	0.0209 (16)	0.045 (2)	−0.0018 (12)	0.0051 (14)	−0.0032 (15)
C29	0.0284 (15)	0.0175 (15)	0.049 (2)	−0.0039 (12)	0.0036 (14)	0.0021 (15)
C30	0.0391 (17)	0.0300 (19)	0.047 (2)	−0.0062 (14)	0.0077 (16)	−0.0074 (17)
C31	0.0277 (16)	0.0317 (19)	0.070 (3)	0.0022 (14)	0.0169 (17)	−0.0070 (19)
C32	0.0290 (15)	0.0192 (16)	0.054 (2)	−0.0006 (12)	−0.0035 (15)	−0.0016 (16)
C33	0.0324 (16)	0.0243 (17)	0.060 (2)	−0.0059 (14)	0.0101 (16)	0.0052 (17)
C34	0.0310 (15)	0.0217 (16)	0.0355 (19)	−0.0044 (12)	0.0032 (14)	−0.0023 (14)
C35	0.0260 (15)	0.0293 (18)	0.044 (2)	0.0036 (13)	−0.0010 (14)	0.0061 (16)
C36	0.0279 (14)	0.0200 (16)	0.0319 (18)	−0.0024 (12)	0.0017 (13)	0.0038 (14)
C37	0.0413 (18)	0.0284 (18)	0.0333 (19)	−0.0011 (14)	0.0083 (15)	0.0053 (15)
C38	0.0249 (14)	0.0253 (17)	0.0388 (19)	0.0015 (12)	0.0022 (13)	0.0016 (15)
C39	0.0340 (16)	0.0301 (18)	0.036 (2)	−0.0040 (14)	0.0019 (14)	−0.0043 (16)
O1W	0.0576 (18)	0.065 (2)	0.087 (3)	0.0022 (16)	0.0022 (17)	−0.0355 (19)
O2W	0.0479 (16)	0.061 (2)	0.0547 (19)	0.0042 (14)	0.0067 (13)	−0.0006 (16)
O4	0.155 (3)	0.054 (2)	0.067 (2)	−0.031 (2)	0.069 (2)	−0.0153 (17)
O3W	0.072 (2)	0.080 (3)	0.072 (3)	−0.0049 (19)	−0.0161 (19)	−0.003 (2)
O5	0.088 (2)	0.079 (2)	0.0488 (18)	−0.0349 (19)	−0.0164 (16)	0.0206 (17)
O4W	0.064 (2)	0.089 (3)	0.098 (3)	−0.003 (2)	0.018 (2)	0.047 (2)
O6	0.088 (2)	0.0288 (15)	0.0622 (19)	−0.0083 (14)	0.0010 (15)	0.0022 (14)
O8	0.0663 (19)	0.076 (3)	0.123 (3)	0.0312 (18)	−0.021 (2)	−0.020 (2)
S1	0.0402 (5)	0.0503 (6)	0.0398 (5)	0.0153 (4)	−0.0062 (4)	−0.0039 (5)
S2	0.0757 (6)	0.0340 (5)	0.0365 (5)	−0.0147 (5)	0.0151 (5)	−0.0004 (4)
S3	0.0599 (6)	0.0515 (7)	0.0640 (7)	0.0196 (5)	−0.0161 (5)	−0.0143 (6)
N1	0.0223 (11)	0.0190 (13)	0.0370 (16)	−0.0010 (10)	0.0041 (11)	−0.0018 (12)
N2	0.0402 (15)	0.0409 (18)	0.0337 (16)	0.0007 (13)	0.0051 (13)	−0.0021 (14)
N3	0.0253 (12)	0.0176 (13)	0.0416 (17)	−0.0008 (10)	0.0059 (11)	0.0019 (12)
N4	0.0200 (11)	0.0194 (13)	0.0393 (16)	−0.0001 (10)	0.0037 (11)	0.0003 (12)
N5	0.0218 (11)	0.0210 (13)	0.0412 (17)	−0.0042 (10)	0.0027 (11)	−0.0024 (12)
N6	0.0364 (14)	0.0339 (17)	0.0500 (19)	−0.0061 (13)	0.0140 (13)	−0.0014 (15)
N7	0.0211 (12)	0.0187 (13)	0.0487 (18)	−0.0009 (10)	−0.0002 (12)	−0.0014 (13)
N8	0.0220 (12)	0.0204 (14)	0.057 (2)	0.0025 (10)	0.0030 (12)	−0.0024 (13)
N9	0.0237 (12)	0.0189 (13)	0.0351 (16)	0.0001 (10)	0.0049 (11)	0.0012 (12)
N10	0.0238 (11)	0.0223 (13)	0.0295 (15)	−0.0006 (10)	0.0011 (11)	−0.0004 (12)
N11	0.0242 (12)	0.0230 (14)	0.0358 (16)	−0.0018 (10)	0.0036 (11)	0.0014 (12)
N12	0.0312 (13)	0.0338 (16)	0.0325 (16)	0.0013 (12)	−0.0016 (12)	0.0067 (13)

### Geometric parameters (Å, °)

**Table:**

Ag1—N1	2.362 (2)	C28—N7	1.483 (4)
Ag1—N9^i^	2.367 (3)	C28—H28A	0.9700
Ag1—N10^ii^	2.388 (3)	C28—H28B	0.9700
Ag2—N5	2.347 (2)	C29—N8	1.478 (4)
Ag2—N7^ii^	2.365 (3)	C29—N5	1.478 (4)
Ag2—N4	2.374 (2)	C29—H29A	0.9700
Ag3—N3^ii^	2.315 (3)	C29—H29B	0.9700
Ag3—N8	2.358 (3)	C30—N6	1.462 (4)
Ag3—N11	2.394 (2)	C30—N5	1.474 (4)
Ag3—O9	2.438 (5)	C30—H30A	0.9700
Ag3—O9'	2.507 (11)	C30—H30B	0.9700
C1—C2	1.380 (5)	C31—N6	1.458 (5)
C1—C6	1.389 (5)	C31—N8	1.479 (5)
C1—S1	1.767 (3)	C31—H31A	0.9700
C2—C3	1.388 (6)	C31—H31B	0.9700
C2—H2	0.9300	C32—N8	1.475 (4)
C3—C4	1.375 (6)	C32—N7	1.477 (4)
C3—H3	0.9300	C32—H32A	0.9700
C4—C5	1.378 (6)	C32—H32B	0.9700
C4—C7	1.494 (6)	C33—N6	1.467 (4)
C5—C6	1.381 (6)	C33—N7	1.480 (4)
C5—H5	0.9300	C33—H33A	0.9700
C6—H6	0.9300	C33—H33B	0.9700
C7—H7A	0.9600	C34—N11	1.477 (4)
C7—H7B	0.9600	C34—N9	1.483 (4)
C7—H7C	0.9600	C34—H34A	0.9700
C8—C9	1.374 (5)	C34—H34B	0.9700
C8—C13	1.385 (5)	C35—N12	1.461 (4)
C8—S3	1.755 (4)	C35—N9	1.483 (4)
C9—C10	1.381 (6)	C35—H35A	0.9700
C9—H9	0.9300	C35—H35B	0.9700
C10—C11	1.379 (7)	C36—N10	1.481 (4)
C10—H10	0.9300	C36—N9	1.489 (4)
C11—C12	1.372 (7)	C36—H36A	0.9700
C11—C14	1.529 (6)	C36—H36B	0.9700
C12—C13	1.388 (6)	C37—N12	1.458 (4)
C12—H12	0.9300	C37—N11	1.479 (4)
C13—H13	0.9300	C37—H37A	0.9700
C14—H14A	0.9600	C37—H37B	0.9700
C14—H14B	0.9600	C38—N10	1.475 (4)
C14—H14C	0.9600	C38—N11	1.477 (4)
C15—C16	1.374 (5)	C38—H38A	0.9700
C15—C20	1.384 (5)	C38—H38B	0.9700
C15—S2	1.769 (4)	C39—N12	1.463 (4)
C16—C17	1.373 (6)	C39—N10	1.477 (4)
C16—H16	0.9300	C39—H39A	0.9700
C17—C18	1.364 (6)	C39—H39B	0.9700
C17—H17	0.9300	O1'—O1	0.837 (7)
C18—C19	1.388 (7)	O1'—S1	1.363 (7)
C18—C21	1.509 (6)	O1—S1	1.469 (5)
C19—C20	1.379 (6)	O2'—O2	0.956 (9)
C19—H19	0.9300	O2'—S1	1.558 (10)
C20—H20	0.9300	O2—S1	1.394 (6)
C21—H21A	0.9600	O2—O3'	1.520 (10)
C21—H21B	0.9600	O1W—H1WA	0.87 (2)
C21—H21C	0.9600	O1W—H1WB	0.82 (2)
C22—N1	1.473 (4)	O3'—O3	1.139 (9)
C22—N3	1.480 (4)	O3'—S1	1.354 (8)
C22—H22A	0.9700	O3—S1	1.504 (6)
C22—H22B	0.9700	O2W—H2WA	0.83 (2)
C23—N2	1.459 (4)	O2W—H2WB	0.84 (2)
C23—N1	1.486 (4)	O4—S2	1.441 (3)
C23—H23A	0.9700	O3W—H3WA	0.80 (2)
C23—H23B	0.9700	O3W—H3WB	0.80 (2)
C24—N2	1.456 (4)	O5—S2	1.446 (3)
C24—N4	1.487 (4)	O4W—H4WA	0.78 (2)
C24—H24A	0.9700	O4W—H4WB	0.88 (2)
C24—H24B	0.9700	O6—S2	1.446 (3)
C25—N4	1.476 (4)	O7'—O7	1.036 (11)
C25—N3	1.485 (4)	O7'—S3	1.628 (12)
C25—H25A	0.9700	O7—S3	1.420 (6)
C25—H25B	0.9700	O7—O9'	1.509 (12)
C26—N4	1.481 (4)	O8—S3	1.389 (3)
C26—N1	1.482 (4)	O9—O9'	1.089 (11)
C26—H26A	0.9700	O9—S3	1.549 (5)
C26—H26B	0.9700	O9'—S3	1.296 (11)
C27—N2	1.460 (5)	N3—Ag3^iii^	2.315 (3)
C27—N3	1.489 (4)	N7—Ag2^iii^	2.365 (3)
C27—H27A	0.9700	N9—Ag1^iv^	2.367 (2)
C27—H27B	0.9700	N10—Ag1^iii^	2.388 (3)
C28—N5	1.474 (4)		
			
N1—Ag1—N9^i^	126.27 (9)	N9—C34—H34B	109.2
N1—Ag1—N10^ii^	109.49 (8)	H34A—C34—H34B	107.9
N9^i^—Ag1—N10^ii^	122.48 (8)	N12—C35—N9	112.4 (2)
N5—Ag2—N7^ii^	125.05 (9)	N12—C35—H35A	109.1
N5—Ag2—N4	115.03 (9)	N9—C35—H35A	109.1
N7^ii^—Ag2—N4	119.83 (8)	N12—C35—H35B	109.1
N3^ii^—Ag3—N8	126.79 (9)	N9—C35—H35B	109.1
N3^ii^—Ag3—N11	113.74 (8)	H35A—C35—H35B	107.9
N8—Ag3—N11	111.58 (9)	N10—C36—N9	111.9 (2)
N3^ii^—Ag3—O9	119.04 (15)	N10—C36—H36A	109.2
N8—Ag3—O9	87.16 (14)	N9—C36—H36A	109.2
N11—Ag3—O9	89.64 (14)	N10—C36—H36B	109.2
N3^ii^—Ag3—O9'	94.9 (3)	N9—C36—H36B	109.2
N8—Ag3—O9'	96.3 (2)	H36A—C36—H36B	107.9
N11—Ag3—O9'	108.0 (3)	N12—C37—N11	112.0 (3)
O9—Ag3—O9'	25.4 (3)	N12—C37—H37A	109.2
C2—C1—C6	119.2 (3)	N11—C37—H37A	109.2
C2—C1—S1	121.3 (3)	N12—C37—H37B	109.2
C6—C1—S1	119.5 (3)	N11—C37—H37B	109.2
C1—C2—C3	119.6 (4)	H37A—C37—H37B	107.9
C1—C2—H2	120.2	N10—C38—N11	112.6 (2)
C3—C2—H2	120.2	N10—C38—H38A	109.1
C4—C3—C2	122.1 (4)	N11—C38—H38A	109.1
C4—C3—H3	119.0	N10—C38—H38B	109.1
C2—C3—H3	119.0	N11—C38—H38B	109.1
C3—C4—C5	117.3 (4)	H38A—C38—H38B	107.8
C3—C4—C7	121.0 (4)	N12—C39—N10	111.9 (3)
C5—C4—C7	121.7 (4)	N12—C39—H39A	109.2
C4—C5—C6	122.2 (4)	N10—C39—H39A	109.2
C4—C5—H5	118.9	N12—C39—H39B	109.2
C6—C5—H5	118.9	N10—C39—H39B	109.2
C5—C6—C1	119.5 (4)	H39A—C39—H39B	107.9
C5—C6—H6	120.2	O1—O1'—S1	79.9 (7)
C1—C6—H6	120.2	O1'—O1—S1	66.0 (6)
C4—C7—H7A	109.5	O2—O2'—S1	62.0 (7)
C4—C7—H7B	109.5	O2'—O2—S1	80.7 (7)
H7A—C7—H7B	109.5	O2'—O2—O3'	135.4 (9)
C4—C7—H7C	109.5	S1—O2—O3'	55.2 (4)
H7A—C7—H7C	109.5	H1WA—O1W—H1WB	107 (3)
H7B—C7—H7C	109.5	O3—O3'—S1	73.6 (6)
C9—C8—C13	119.8 (4)	O3—O3'—O2	127.6 (7)
C9—C8—S3	120.6 (3)	S1—O3'—O2	57.7 (4)
C13—C8—S3	119.4 (3)	O3'—O3—S1	59.8 (5)
C8—C9—C10	119.4 (4)	H2WA—O2W—H2WB	109 (3)
C8—C9—H9	120.3	H3WA—O3W—H3WB	113 (4)
C10—C9—H9	120.3	H4WA—O4W—H4WB	104 (3)
C11—C10—C9	121.3 (4)	O7—O7'—S3	59.6 (7)
C11—C10—H10	119.4	O7'—O7—S3	81.4 (8)
C9—C10—H10	119.4	O7'—O7—O9'	130.5 (10)
C12—C11—C10	119.0 (4)	S3—O7—O9'	52.4 (5)
C12—C11—C14	119.9 (6)	O9'—O9—S3	55.6 (6)
C10—C11—C14	121.1 (5)	O9'—O9—Ag3	80.9 (7)
C11—C12—C13	120.3 (5)	S3—O9—Ag3	134.4 (3)
C11—C12—H12	119.9	O9—O9'—S3	80.5 (8)
C13—C12—H12	119.9	O9—O9'—O7	140.3 (10)
C8—C13—C12	120.0 (4)	S3—O9'—O7	60.2 (5)
C8—C13—H13	120.0	O9—O9'—Ag3	73.7 (6)
C12—C13—H13	120.0	S3—O9'—Ag3	150.6 (7)
C11—C14—H14A	109.5	O7—O9'—Ag3	141.3 (7)
C11—C14—H14B	109.5	O3'—S1—O1'	122.4 (5)
H14A—C14—H14B	109.5	O3'—S1—O2	67.1 (4)
C11—C14—H14C	109.5	O1'—S1—O2	136.8 (4)
H14A—C14—H14C	109.5	O3'—S1—O1	143.2 (4)
H14B—C14—H14C	109.5	O1'—S1—O1	34.1 (3)
C16—C15—C20	119.6 (4)	O2—S1—O1	110.5 (3)
C16—C15—S2	121.0 (3)	O3'—S1—O3	46.6 (4)
C20—C15—S2	119.4 (3)	O1'—S1—O3	80.0 (4)
C17—C16—C15	120.0 (4)	O2—S1—O3	111.2 (3)
C17—C16—H16	120.0	O1—S1—O3	111.8 (3)
C15—C16—H16	120.0	O3'—S1—O2'	104.2 (5)
C18—C17—C16	121.5 (4)	O1'—S1—O2'	111.7 (5)
C18—C17—H17	119.2	O2—S1—O2'	37.3 (3)
C16—C17—H17	119.2	O1—S1—O2'	78.6 (4)
C17—C18—C19	118.4 (4)	O3—S1—O2'	144.1 (4)
C17—C18—C21	121.7 (5)	O3'—S1—C1	108.2 (4)
C19—C18—C21	119.8 (5)	O1'—S1—C1	107.0 (3)
C20—C19—C18	120.9 (4)	O2—S1—C1	108.5 (3)
C20—C19—H19	119.6	O1—S1—C1	107.1 (2)
C18—C19—H19	119.6	O3—S1—C1	107.5 (2)
C19—C20—C15	119.6 (4)	O2'—S1—C1	101.2 (4)
C19—C20—H20	120.2	O4—S2—O5	112.7 (2)
C15—C20—H20	120.2	O4—S2—O6	112.10 (18)
C18—C21—H21A	109.5	O5—S2—O6	112.3 (2)
C18—C21—H21B	109.5	O4—S2—C15	105.4 (2)
H21A—C21—H21B	109.5	O5—S2—C15	106.32 (17)
C18—C21—H21C	109.5	O6—S2—C15	107.46 (17)
H21A—C21—H21C	109.5	O9'—S3—O8	126.5 (5)
H21B—C21—H21C	109.5	O9'—S3—O7	67.3 (6)
N1—C22—N3	112.0 (2)	O8—S3—O7	122.3 (3)
N1—C22—H22A	109.2	O9'—S3—O9	43.9 (5)
N3—C22—H22A	109.2	O8—S3—O9	104.8 (3)
N1—C22—H22B	109.2	O7—S3—O9	111.0 (3)
N3—C22—H22B	109.2	O9'—S3—O7'	104.5 (7)
H22A—C22—H22B	107.9	O8—S3—O7'	90.8 (5)
N2—C23—N1	111.8 (3)	O7—S3—O7'	39.0 (4)
N2—C23—H23A	109.2	O9—S3—O7'	147.9 (5)
N1—C23—H23A	109.2	O9'—S3—C8	116.8 (4)
N2—C23—H23B	109.2	O8—S3—C8	108.9 (2)
N1—C23—H23B	109.2	O7—S3—C8	108.6 (3)
H23A—C23—H23B	107.9	O9—S3—C8	98.7 (2)
N2—C24—N4	111.3 (2)	O7'—S3—C8	102.5 (4)
N2—C24—H24A	109.4	C22—N1—C26	108.0 (2)
N4—C24—H24A	109.4	C22—N1—C23	107.7 (3)
N2—C24—H24B	109.4	C26—N1—C23	108.4 (2)
N4—C24—H24B	109.4	C22—N1—Ag1	108.63 (17)
H24A—C24—H24B	108.0	C26—N1—Ag1	108.22 (18)
N4—C25—N3	111.5 (2)	C23—N1—Ag1	115.60 (18)
N4—C25—H25A	109.3	C24—N2—C23	109.5 (3)
N3—C25—H25A	109.3	C24—N2—C27	108.7 (3)
N4—C25—H25B	109.3	C23—N2—C27	108.8 (3)
N3—C25—H25B	109.3	C22—N3—C25	108.1 (2)
H25A—C25—H25B	108.0	C22—N3—C27	108.0 (2)
N4—C26—N1	111.6 (2)	C25—N3—C27	107.9 (2)
N4—C26—H26A	109.3	C22—N3—Ag3^iii^	108.95 (17)
N1—C26—H26A	109.3	C25—N3—Ag3^iii^	108.15 (18)
N4—C26—H26B	109.3	C27—N3—Ag3^iii^	115.50 (19)
N1—C26—H26B	109.3	C25—N4—C26	107.6 (2)
H26A—C26—H26B	108.0	C25—N4—C24	108.7 (3)
N2—C27—N3	111.7 (3)	C26—N4—C24	108.8 (2)
N2—C27—H27A	109.3	C25—N4—Ag2	113.34 (17)
N3—C27—H27A	109.3	C26—N4—Ag2	104.51 (18)
N2—C27—H27B	109.3	C24—N4—Ag2	113.63 (18)
N3—C27—H27B	109.3	C30—N5—C28	107.9 (2)
H27A—C27—H27B	107.9	C30—N5—C29	108.7 (2)
N5—C28—N7	112.2 (2)	C28—N5—C29	108.7 (2)
N5—C28—H28A	109.2	C30—N5—Ag2	113.08 (18)
N7—C28—H28A	109.2	C28—N5—Ag2	109.63 (16)
N5—C28—H28B	109.2	C29—N5—Ag2	108.77 (18)
N7—C28—H28B	109.2	C31—N6—C30	108.2 (3)
H28A—C28—H28B	107.9	C31—N6—C33	108.7 (3)
N8—C29—N5	111.4 (2)	C30—N6—C33	108.7 (2)
N8—C29—H29A	109.4	C32—N7—C33	107.9 (2)
N5—C29—H29A	109.4	C32—N7—C28	107.5 (2)
N8—C29—H29B	109.4	C33—N7—C28	107.4 (3)
N5—C29—H29B	109.4	C32—N7—Ag2^iii^	107.44 (19)
H29A—C29—H29B	108.0	C33—N7—Ag2^iii^	116.58 (19)
N6—C30—N5	111.6 (3)	C28—N7—Ag2^iii^	109.70 (17)
N6—C30—H30A	109.3	C32—N8—C29	107.9 (2)
N5—C30—H30A	109.3	C32—N8—C31	108.6 (3)
N6—C30—H30B	109.3	C29—N8—C31	107.4 (3)
N5—C30—H30B	109.3	C32—N8—Ag3	106.1 (2)
H30A—C30—H30B	108.0	C29—N8—Ag3	110.9 (2)
N6—C31—N8	112.4 (2)	C31—N8—Ag3	115.70 (18)
N6—C31—H31A	109.1	C34—N9—C35	107.7 (2)
N8—C31—H31A	109.1	C34—N9—C36	107.9 (2)
N6—C31—H31B	109.1	C35—N9—C36	107.8 (2)
N8—C31—H31B	109.1	C34—N9—Ag1^iv^	106.83 (18)
H31A—C31—H31B	107.9	C35—N9—Ag1^iv^	117.58 (17)
N8—C32—N7	112.6 (3)	C36—N9—Ag1^iv^	108.72 (18)
N8—C32—H32A	109.1	C38—N10—C39	108.0 (2)
N7—C32—H32A	109.1	C38—N10—C36	107.7 (2)
N8—C32—H32B	109.1	C39—N10—C36	108.3 (2)
N7—C32—H32B	109.1	C38—N10—Ag1^iii^	111.35 (17)
H32A—C32—H32B	107.8	C39—N10—Ag1^iii^	114.17 (19)
N6—C33—N7	112.3 (3)	C36—N10—Ag1^iii^	107.13 (18)
N6—C33—H33A	109.1	C34—N11—C38	107.6 (2)
N7—C33—H33A	109.1	C34—N11—C37	108.5 (2)
N6—C33—H33B	109.1	C38—N11—C37	107.9 (2)
N7—C33—H33B	109.1	C34—N11—Ag3	109.81 (18)
H33A—C33—H33B	107.9	C38—N11—Ag3	109.83 (16)
N11—C34—N9	111.9 (2)	C37—N11—Ag3	113.03 (18)
N11—C34—H34A	109.2	C37—N12—C35	108.2 (3)
N9—C34—H34A	109.2	C37—N12—C39	109.0 (2)
N11—C34—H34B	109.2	C35—N12—C39	108.9 (3)
			
C6—C1—C2—C3	0.2 (6)	O7—O7'—S3—O9	26.1 (12)
S1—C1—C2—C3	178.8 (3)	O7—O7'—S3—C8	−104.2 (6)
C1—C2—C3—C4	0.0 (6)	C9—C8—S3—O9'	−54.3 (7)
C2—C3—C4—C5	−0.5 (6)	C13—C8—S3—O9'	121.2 (6)
C2—C3—C4—C7	179.1 (4)	C9—C8—S3—O8	154.4 (3)
C3—C4—C5—C6	0.8 (7)	C13—C8—S3—O8	−30.1 (4)
C7—C4—C5—C6	−178.8 (4)	C9—C8—S3—O7	19.1 (4)
C4—C5—C6—C1	−0.5 (7)	C13—C8—S3—O7	−165.4 (4)
C2—C1—C6—C5	0.0 (6)	C9—C8—S3—O9	−96.6 (4)
S1—C1—C6—C5	−178.6 (3)	C13—C8—S3—O9	78.9 (4)
C13—C8—C9—C10	−2.0 (5)	C9—C8—S3—O7'	59.2 (5)
S3—C8—C9—C10	173.5 (3)	C13—C8—S3—O7'	−125.3 (5)
C8—C9—C10—C11	−1.2 (6)	N3—C22—N1—C26	58.5 (3)
C9—C10—C11—C12	3.7 (6)	N3—C22—N1—C23	−58.4 (3)
C9—C10—C11—C14	−175.8 (4)	N3—C22—N1—Ag1	175.7 (2)
C10—C11—C12—C13	−3.1 (7)	N4—C26—N1—C22	−59.4 (3)
C14—C11—C12—C13	176.4 (4)	N4—C26—N1—C23	57.0 (3)
C9—C8—C13—C12	2.5 (6)	N4—C26—N1—Ag1	−176.8 (2)
S3—C8—C13—C12	−173.0 (3)	N2—C23—N1—C22	59.0 (3)
C11—C12—C13—C8	0.0 (6)	N2—C23—N1—C26	−57.7 (3)
C20—C15—C16—C17	1.0 (6)	N2—C23—N1—Ag1	−179.3 (2)
S2—C15—C16—C17	179.0 (3)	N9^i^—Ag1—N1—C22	−22.2 (2)
C15—C16—C17—C18	−0.2 (6)	N10^ii^—Ag1—N1—C22	172.70 (19)
C16—C17—C18—C19	−0.3 (7)	N9^i^—Ag1—N1—C26	94.8 (2)
C16—C17—C18—C21	177.1 (4)	N10^ii^—Ag1—N1—C26	−70.3 (2)
C17—C18—C19—C20	−0.1 (7)	N9^i^—Ag1—N1—C23	−143.4 (2)
C21—C18—C19—C20	−177.5 (4)	N10^ii^—Ag1—N1—C23	51.5 (2)
C18—C19—C20—C15	0.8 (6)	N4—C24—N2—C23	−59.0 (4)
C16—C15—C20—C19	−1.3 (6)	N4—C24—N2—C27	59.8 (3)
S2—C15—C20—C19	−179.4 (3)	N1—C23—N2—C24	59.1 (4)
S1—O2'—O2—O3'	7.9 (10)	N1—C23—N2—C27	−59.7 (3)
O2'—O2—O3'—O3	15.1 (17)	N3—C27—N2—C24	−60.2 (3)
S1—O2—O3'—O3	24.6 (8)	N3—C27—N2—C23	59.0 (3)
O2'—O2—O3'—S1	−9.5 (12)	N1—C22—N3—C25	−58.4 (3)
O2—O3'—O3—S1	−21.5 (6)	N1—C22—N3—C27	58.1 (3)
S3—O7'—O7—O9'	−19.8 (9)	N1—C22—N3—Ag3^iii^	−175.7 (2)
N3^ii^—Ag3—O9—O9'	−20.0 (6)	N4—C25—N3—C22	59.0 (3)
N8—Ag3—O9—O9'	111.2 (6)	N4—C25—N3—C27	−57.6 (3)
N11—Ag3—O9—O9'	−137.2 (6)	N4—C25—N3—Ag3^iii^	176.85 (19)
N3^ii^—Ag3—O9—S3	−3.3 (4)	N2—C27—N3—C22	−58.0 (3)
N8—Ag3—O9—S3	127.9 (4)	N2—C27—N3—C25	58.7 (3)
N11—Ag3—O9—S3	−120.5 (4)	N2—C27—N3—Ag3^iii^	179.78 (19)
O9'—Ag3—O9—S3	16.7 (5)	N3—C25—N4—C26	−59.8 (3)
Ag3—O9—O9'—S3	165.5 (4)	N3—C25—N4—C24	57.9 (3)
S3—O9—O9'—O7	−8.3 (10)	N3—C25—N4—Ag2	−174.78 (18)
Ag3—O9—O9'—O7	157.3 (14)	N1—C26—N4—C25	60.1 (3)
S3—O9—O9'—Ag3	−165.5 (4)	N1—C26—N4—C24	−57.5 (3)
O7'—O7—O9'—O9	34 (2)	N1—C26—N4—Ag2	−179.2 (2)
S3—O7—O9'—O9	9.4 (12)	N2—C24—N4—C25	−58.8 (3)
O7'—O7—O9'—S3	25.1 (11)	N2—C24—N4—C26	58.1 (3)
O7'—O7—O9'—Ag3	178.1 (10)	N2—C24—N4—Ag2	174.1 (2)
S3—O7—O9'—Ag3	153.0 (11)	N5—Ag2—N4—C25	−39.8 (2)
N3^ii^—Ag3—O9'—O9	162.5 (6)	N7^ii^—Ag2—N4—C25	136.98 (19)
N8—Ag3—O9'—O9	−69.6 (6)	N5—Ag2—N4—C26	−156.63 (18)
N11—Ag3—O9'—O9	45.6 (6)	N7^ii^—Ag2—N4—C26	20.2 (2)
N3^ii^—Ag3—O9'—S3	132.5 (14)	N5—Ag2—N4—C24	84.9 (2)
N8—Ag3—O9'—S3	−99.6 (14)	N7^ii^—Ag2—N4—C24	−98.3 (2)
N11—Ag3—O9'—S3	15.5 (15)	N6—C30—N5—C28	59.2 (3)
O9—Ag3—O9'—S3	−30.1 (10)	N6—C30—N5—C29	−58.5 (3)
N3^ii^—Ag3—O9'—O7	5.8 (10)	N6—C30—N5—Ag2	−179.4 (2)
N8—Ag3—O9'—O7	133.7 (9)	N7—C28—N5—C30	−59.2 (3)
N11—Ag3—O9'—O7	−111.2 (9)	N7—C28—N5—C29	58.5 (3)
O9—Ag3—O9'—O7	−156.8 (14)	N7—C28—N5—Ag2	177.2 (2)
O3—O3'—S1—O1'	−27.9 (7)	N8—C29—N5—C30	58.5 (3)
O2—O3'—S1—O1'	132.0 (5)	N8—C29—N5—C28	−58.7 (3)
O3—O3'—S1—O2	−159.9 (6)	N8—C29—N5—Ag2	−178.0 (2)
O3—O3'—S1—O1	−65.9 (9)	N7^ii^—Ag2—N5—C30	115.7 (2)
O2—O3'—S1—O1	94.1 (7)	N4—Ag2—N5—C30	−67.7 (2)
O2—O3'—S1—O3	159.9 (6)	N7^ii^—Ag2—N5—C28	−123.9 (2)
O3—O3'—S1—O2'	−155.7 (5)	N4—Ag2—N5—C28	52.7 (2)
O2—O3'—S1—O2'	4.2 (5)	N7^ii^—Ag2—N5—C29	−5.1 (2)
O3—O3'—S1—C1	97.2 (5)	N4—Ag2—N5—C29	171.48 (19)
O2—O3'—S1—C1	−102.9 (3)	N8—C31—N6—C30	−59.7 (4)
O1—O1'—S1—O3'	−138.9 (7)	N8—C31—N6—C33	58.1 (3)
O1—O1'—S1—O2	−48.7 (9)	N5—C30—N6—C31	58.7 (4)
O1—O1'—S1—O3	−159.1 (7)	N5—C30—N6—C33	−59.1 (4)
O1—O1'—S1—O2'	−14.5 (8)	N7—C33—N6—C31	−58.9 (3)
O1—O1'—S1—C1	95.5 (6)	N7—C33—N6—C30	58.7 (4)
O2'—O2—S1—O3'	173.3 (8)	N8—C32—N7—C33	−57.1 (3)
O2'—O2—S1—O1'	59.7 (9)	N8—C32—N7—C28	58.5 (3)
O3'—O2—S1—O1'	−113.6 (6)	N8—C32—N7—Ag2^iii^	176.5 (2)
O2'—O2—S1—O1	32.9 (7)	N6—C33—N7—C32	58.0 (3)
O3'—O2—S1—O1	−140.3 (4)	N6—C33—N7—C28	−57.6 (3)
O2'—O2—S1—O3	157.7 (7)	N6—C33—N7—Ag2^iii^	178.89 (19)
O3'—O2—S1—O3	−15.5 (5)	N5—C28—N7—C32	−57.7 (4)
O3'—O2—S1—O2'	−173.3 (8)	N5—C28—N7—C33	58.2 (3)
O2'—O2—S1—C1	−84.2 (7)	N5—C28—N7—Ag2^iii^	−174.2 (2)
O3'—O2—S1—C1	102.5 (4)	N7—C32—N8—C29	−59.4 (4)
O1'—O1—S1—O3'	67.8 (10)	N7—C32—N8—C31	56.7 (3)
O1'—O1—S1—O2	146.7 (6)	N7—C32—N8—Ag3	−178.3 (2)
O1'—O1—S1—O3	22.2 (7)	N5—C29—N8—C32	58.7 (4)
O1'—O1—S1—O2'	166.3 (7)	N5—C29—N8—C31	−58.2 (3)
O1'—O1—S1—C1	−95.3 (6)	N5—C29—N8—Ag3	174.5 (2)
O3'—O3—S1—O1'	156.3 (6)	N6—C31—N8—C32	−57.1 (3)
O3'—O3—S1—O2	19.8 (6)	N6—C31—N8—C29	59.4 (3)
O3'—O3—S1—O1	143.9 (5)	N6—C31—N8—Ag3	−176.2 (2)
O3'—O3—S1—O2'	42.9 (9)	N3^ii^—Ag3—N8—C32	145.73 (18)
O3'—O3—S1—C1	−98.8 (5)	N11—Ag3—N8—C32	−67.5 (2)
O2—O2'—S1—O3'	−6.4 (8)	O9—Ag3—N8—C32	21.0 (2)
O2—O2'—S1—O1'	−140.5 (6)	O9'—Ag3—N8—C32	44.7 (3)
O2—O2'—S1—O1	−148.7 (7)	N3^ii^—Ag3—N8—C29	28.8 (2)
O2—O2'—S1—O3	−37.1 (11)	N11—Ag3—N8—C29	175.6 (2)
O2—O2'—S1—C1	105.9 (6)	O9—Ag3—N8—C29	−95.9 (2)
C2—C1—S1—O3'	−3.4 (5)	O9'—Ag3—N8—C29	−72.2 (3)
C6—C1—S1—O3'	175.2 (5)	N3^ii^—Ag3—N8—C31	−93.7 (2)
C2—C1—S1—O1'	130.3 (4)	N11—Ag3—N8—C31	53.0 (2)
C6—C1—S1—O1'	−51.1 (5)	O9—Ag3—N8—C31	141.5 (2)
C2—C1—S1—O2	−74.7 (4)	O9'—Ag3—N8—C31	165.2 (3)
C6—C1—S1—O2	103.9 (4)	N11—C34—N9—C35	57.2 (3)
C2—C1—S1—O1	166.0 (4)	N11—C34—N9—C36	−59.0 (3)
C6—C1—S1—O1	−15.4 (4)	N11—C34—N9—Ag1^iv^	−175.7 (2)
C2—C1—S1—O3	45.7 (4)	N12—C35—N9—C34	−58.5 (3)
C6—C1—S1—O3	−135.7 (4)	N12—C35—N9—C36	57.6 (3)
C2—C1—S1—O2'	−112.6 (5)	N12—C35—N9—Ag1^iv^	−179.08 (19)
C6—C1—S1—O2'	66.0 (5)	N10—C36—N9—C34	58.6 (3)
C16—C15—S2—O4	−109.5 (3)	N10—C36—N9—C35	−57.4 (3)
C20—C15—S2—O4	68.6 (3)	N10—C36—N9—Ag1^iv^	174.05 (17)
C16—C15—S2—O5	10.3 (4)	N11—C38—N10—C39	−57.6 (3)
C20—C15—S2—O5	−171.6 (3)	N11—C38—N10—C36	59.1 (3)
C16—C15—S2—O6	130.8 (3)	N11—C38—N10—Ag1^iii^	176.3 (2)
C20—C15—S2—O6	−51.1 (3)	N12—C39—N10—C38	57.8 (3)
O9—O9'—S3—O8	71.8 (8)	N12—C39—N10—C36	−58.6 (3)
O7—O9'—S3—O8	−114.3 (5)	N12—C39—N10—Ag1^iii^	−177.78 (18)
Ag3—O9'—S3—O8	101.0 (13)	N9—C36—N10—C38	−58.4 (3)
O9—O9'—S3—O7	−173.9 (8)	N9—C36—N10—C39	58.1 (3)
Ag3—O9'—S3—O7	−144.8 (15)	N9—C36—N10—Ag1^iii^	−178.29 (18)
O7—O9'—S3—O9	173.9 (8)	N9—C34—N11—C38	59.0 (3)
Ag3—O9'—S3—O9	29.2 (10)	N9—C34—N11—C37	−57.5 (3)
O9—O9'—S3—O7'	173.8 (6)	N9—C34—N11—Ag3	178.53 (19)
O7—O9'—S3—O7'	−12.2 (6)	N10—C38—N11—C34	−59.3 (3)
Ag3—O9'—S3—O7'	−157.0 (13)	N10—C38—N11—C37	57.6 (3)
O9—O9'—S3—C8	−73.8 (7)	N10—C38—N11—Ag3	−178.8 (2)
O7—O9'—S3—C8	100.2 (4)	N12—C37—N11—C34	58.4 (3)
Ag3—O9'—S3—C8	−44.6 (15)	N12—C37—N11—C38	−57.9 (3)
O7'—O7—S3—O9'	−161.0 (9)	N12—C37—N11—Ag3	−179.5 (2)
O7'—O7—S3—O8	−41.1 (8)	N3^ii^—Ag3—N11—C34	66.1 (2)
O9'—O7—S3—O8	119.9 (5)	N8—Ag3—N11—C34	−85.2 (2)
O7'—O7—S3—O9	−165.5 (7)	O9—Ag3—N11—C34	−172.0 (2)
O9'—O7—S3—O9	−4.5 (6)	O9'—Ag3—N11—C34	170.1 (3)
O9'—O7—S3—O7'	161.0 (9)	N3^ii^—Ag3—N11—C38	−175.77 (19)
O7'—O7—S3—C8	87.0 (7)	N8—Ag3—N11—C38	32.9 (2)
O9'—O7—S3—C8	−112.0 (5)	O9—Ag3—N11—C38	−53.9 (2)
Ag3—O9—S3—O9'	−20.2 (6)	O9'—Ag3—N11—C38	−71.8 (3)
O9'—O9—S3—O8	−127.9 (7)	N3^ii^—Ag3—N11—C37	−55.2 (2)
Ag3—O9—S3—O8	−148.0 (3)	N8—Ag3—N11—C37	153.4 (2)
O9'—O9—S3—O7	6.0 (7)	O9—Ag3—N11—C37	66.6 (2)
Ag3—O9—S3—O7	−14.2 (5)	O9'—Ag3—N11—C37	48.8 (3)
O9'—O9—S3—O7'	−11.3 (11)	N11—C37—N12—C35	−59.2 (3)
Ag3—O9—S3—O7'	−31.5 (10)	N11—C37—N12—C39	59.1 (3)
O9'—O9—S3—C8	119.9 (7)	N9—C35—N12—C37	59.7 (3)
Ag3—O9—S3—C8	99.7 (4)	N9—C35—N12—C39	−58.6 (3)
O7—O7'—S3—O9'	18.1 (8)	N10—C39—N12—C37	−59.0 (3)
O7—O7'—S3—O8	146.3 (6)	N10—C39—N12—C35	58.8 (3)

Symmetry codes: (i) *x*−1, *y*, *z*; (ii) −*x*+3/2, *y*−1/2, −*z*+3/2; (iii) −*x*+3/2, *y*+1/2, −*z*+3/2; (iv) *x*+1, *y*, *z*.

### Hydrogen-bond geometry (Å, °)

**Table:**

*D*—H···*A*	*D*—H	H···*A*	*D*···*A*	*D*—H···*A*
O1W—H1WA···O8^ii^	0.87 (2)	1.92 (2)	2.782 (5)	175 (4)
O1W—H1WB···O6^ii^	0.82 (2)	2.10 (2)	2.907 (4)	166 (4)
O2W—H2WA···O5^iii^	0.83 (2)	2.16 (2)	2.958 (4)	163 (4)
O2W—H2WB···O1^iv^	0.84 (2)	1.83 (3)	2.612 (6)	155 (4)
O3W—H3WA···O2^v^	0.80 (2)	2.45 (3)	3.096 (7)	139 (4)
O3W—H3WB···O5	0.80 (2)	2.15 (2)	2.908 (5)	160 (4)
O4W—H4WB···O7^iii^	0.88 (2)	1.99 (3)	2.838 (7)	160 (4)

Symmetry codes: (ii) −*x*+3/2, *y*−1/2, −*z*+3/2; (iii) −*x*+3/2, *y*+1/2, −*z*+3/2; (iv) *x*+1, *y*, *z*; (v) −*x*+1/2, *y*+1/2, −*z*+3/2.
